# Relationship between plasma urea and copeptin in response to arginine stimulation in healthy adults, patients with vasopressin deficiency and primary polydipsia

**DOI:** 10.1007/s11102-024-01489-7

**Published:** 2025-01-25

**Authors:** Cihan Atila, Sven Lustenberger, Irina Chifu, Emanuele Ferrante, Zoran Erlic, Juliana B. Drummond, Rita Indirli, Roosmarijn Drexhage, Andrew S. Powlson, Mark Gurnell, Beatriz Santana Soares, Johannes Hofland, Felix Beuschlein, Martin Fassnacht, Bettina Winzeler, Julie Refardt, Mirjam Christ-Crain

**Affiliations:** 1 Departments of Endocrinology, Diabetology and Metabolism, University Hospital Basel, Petersgraben 4, 4031 Basel, Switzerland; 2 Department of Clinical Research, University Hospital Basel, University of Basel, Basel, Switzerland; 3 Section of Endocrinology, Department of Internal Medicine, Erasmus Medical Center, Rotterdam, The Netherlands; 4 Division of Endocrinology and Diabetes, Department of Internal Medicine I, University Hospital, University of Wuerzburg, Wurzburg, Germany; 5 Endocrinology Unit, Fondazione IRCCS Ca’Granda Ospedale Maggiore Policlinico, Milan, Italy; 6 Department of Endocrinology, Diabetology, and Clinical Nutrition, University Hospital Zurich (USZ) and University of Zurich (UZH), Zurich, Switzerland; 7 Department of Internal Medicine, Medical School of the Federal University of Minas Gerais, Belo Horizonte, Minas Gerais Brazil; 8 Department of Clinical Sciences and Community Health, University of Milan, Milan, Italy; 9 Institute of Metabolic Science, University of Cambridge and Addenbrooke’s Hospital, Cambridge Biomedical Campus, Cambridge, UK; 10 Cambridge NIHR Biomedical Research Centre, Cambridge University Hospitals, Cambridge, UK; 11 Medizinische Klinik und Poliklinik IV, Klinikum der Universität, Ludwig-Maximilians-Universität München, Munich, Germany; 12 Central Laboratory, University Hospital Wuerzburg, Wurzburg, Germany; 13 The LOOP Zurich—Medical Research Center, Zurich, Switzerland

**Keywords:** Arginine vasopressin, Posterior pituitary, Stimulation test, Polyuria polydipsia, Diabetes insipidus, Pituitary

## Abstract

**Background:**

Arginine infusion stimulates copeptin secretion, a surrogate marker of arginine vasopressin (AVP), thereby serving as a diagnostic test in the differential diagnosis of suspected AVP deficiency (AVP-D). Yet, the precise mechanism underlying the stimulatory effect of arginine on the vasopressinergic system remains elusive. Arginine plays a significant role in the urea cycle and increases the production of urea. An increase in plasma urea concentration raises blood osmolality, thereby possibly stimulating AVP release. We therefore hypothesized that the stimulatory effect of arginine on AVP may involve an increase in plasma urea levels.

**Methods:**

This analysis combined data from two prospective diagnostic studies. In total, 30 healthy adults (HA), 69 patients with AVP-D, and 89 patients with primary polydipsia (PP) underwent the arginine stimulation test. Infusion of arginine (L-­arginine­-hydrochloride 21%) at a dose of 0.5 g/kg body weight diluted in 500 mL of 0.9% normal saline was administered over 30 min. Blood was collected at baseline and 60, 90, and 120 min to analyze plasma copeptin and urea. The main objective was to investigate urea dynamics in response to arginine administration and its effect on copeptin release.

**Results:**

Plasma urea levels at baseline were comparable and increased 60 min after arginine infusion with a median (IQR) change of + 1.1 mmol/L (+ 0.8, + 1.5) in HA, + 1.4 mmol/L (+ 1.1, + 1.7) in patients with AVP-D and + 1.3 mmol/L (+ 0.9, + 1.5) in patients with PP. Concurrently, plasma copeptin levels substantially increased 60 min from baseline in HA (median change + 5.3 pmol/L (+ 3.2, + 8.8)) and in patients with PP (median change + 2.4 pmol/L (+ 1.2, + 3.8)), but remained stable in patients with AVP-D (median change + 0.3 pmol/L (+ 0.1, + 0.6)). Plasma urea and copeptin levels correlated the most in HA, with a Spearman’s rho of 0.41 at baseline. Patients with AVP-D and PP showed only weak correlations of plasma urea and copeptin, with a correlation coefficient between 0.01 and 0.28.

**Conclusion:**

We demonstrate a slight increase in plasma urea levels in response to arginine, but plasma urea and copeptin levels were weakly correlated. Based on these findings, the stimulatory effect of arginine on AVP cannot be explained primarily by increasing urea levels.

## Introduction

Arginine vasopressin (AVP) is a peptide hormone synthesized in the hypothalamus and released into circulation from the posterior pituitary [1]. The main stimulus for AVP release is an increase in plasma osmolality [2]. Copeptin is the C-terminal segment of the AVP precursor peptide and secreted equimolar to AVP, making it a valuable surrogate marker in clinical settings for diagnosing conditions related to AVP dysregulation [3].

The diagnostic gold standard in case of suspected AVP deficiency (AVP-D) is osmotically-stimulated copeptin measurement using hypertonic saline infusion [4–6]. As an alternative approach, several studies have demonstrated that the administration of arginine, a conditionally essential amino acid, has the potential to stimulate the vasopressinergic system [7–9]. Specifically, initial research findings have suggested that infusing arginine in healthy adults (HA) leads to an elevation in plasma copeptin [7]. Subsequent studies have investigated this effect as a diagnostic test, revealing that patients with AVP-D show no or little increase in copeptin in response to arginine, while in patients with primary polydipsia (PP) a substantial increase in copeptin was observed [6–9]. However, the precise mechanism underlying this stimulatory effect of arginine on AVP remains unknown. Arginine plays a significant role in the urea cycle, a metabolic pathway resulting in the conversion of toxic ammonia into urea [10]. Within this cycle, arginine is converted into urea and ornithine, thus higher levels of plasma arginine favor the production of urea. Urea contributes to overall plasma osmolality [11], and it has been demonstrated previously that an increase in plasma osmolality due to intravenous infusion of urea increases the secretion of AVP [12].

We hypothesized that the effect of arginine on AVP may involve an increase in plasma urea levels, thus rising plasma osmolality, acting as an osmotic stimulus for AVP release. We therefore investigated plasma urea and osmolality dynamics in response to arginine stimulation and assessed the effect of plasma urea levels on plasma copeptin in HA, patients with AVP-D and PP.

## Material and methods

### Study design and participants

This study combined data from two prospective diagnostic studies [6, 7]. The first study was conducted at the University Hospital Basel in Switzerland. In total, 30 HA received a single intravenous weight-adapted dose of arginine. Inclusion criteria for HA were age 18 years or older, a body mass index (BMI) between 18.5 and 25 kg/m^2^, normal drinking habits defined as fluid intake of less than 3 L/d, and no history of polyuria. The second study was a prospective international multicenter study conducted at seven tertiary medical centers in Switzerland, Germany, the Netherlands, Italy, the United Kingdom, and Brazil. In total, 69 patients with AVP-D and 89 PP received a single intravenous weight-adapted dose of arginine. Inclusion criteria were age over 18 years, polydipsia (> 3 L/day) and hypotonic polyuria (> 50 mL/kg body weight in 24 h urine collection and urine osmolality < 800 mOsm/kg) or patients with a previously confirmed diagnosis of AVP-D due to any etiology. Exclusion criteria were AVP resistance, polyuria-polydipsia due to other causes (diabetes mellitus, hypokalemia, or hypercalcemia), uncontrolled adrenal or thyroid hormone deficiency, treatment for epilepsy, uncontrolled arterial hypertension, heart failure, liver cirrhosis, pregnancy or breastfeeding or any relevant acute or terminal illness.

Patients treated with desmopressin stopped their medication 24 h before the test, or at least 12 h before the test in case of severe polyuria and/or polydipsia. Patients with known adrenal insufficiency received a standardized stress dose of 50 mg hydrocortisone on the test day. Full details of the studies’ rationale, design, test protocol, and statistical analysis have been published elsewhere [6, 7]. The local ethics committee approved the study protocols. Written informed consent was obtained from all study participants. The studies were registered on ClinicalTrials.gov, identifier NCT 01879137 and NCT 03572166.

### Arginine infusion test protocol

Participants presented between 08:00 and 10:00 a.m. after an overnight fast of 8 h and fluid restriction of 2 h. A catheter was placed into an antecubital vein. A first blood sample was collected at baseline immediately before starting the infusion. The infusion of arginine (L­-arginine­-hydrochloride 21%, Braun, B Braun Melsungen AG, Melsungen, Germany) was infused over 30 min at a dose of 0.5 g/kg body weight, with a maximum of 40 g, diluted in 500 mL of 0.9% saline. Participants were not allowed to drink or eat during the test. For this analysis, we analyzed blood samples taken for copeptin, osmolality, and urea measurement at baseline, 60 and 120 min in HA. In healthy adults, the peak of copeptin was observed at 60 min. Therefore, in patients with PP and AVP-D, the test protocol was shortened to 90 min and blood was taken at baseline, 60 and 90 min.

### Laboratory measurements

Plasma samples for copeptin, osmolality, and urea were immediately centrifuged at 4 °C and stored at − 80 °C until central batch analysis. Plasma copeptin concentration upon arginine stimulation was measured in one batch with a commercial automated immunofluorescence assay (B.R.A.H.M.S Copeptin-proAVP KRYPTOR, Thermo Scientific Biomarkers, Hennigsdorf, Germany). Plasma urea, sodium, glucose, and osmolality were measured in the laboratory immediately after collection, except for the 60 min values in HA, which were measured in batch-analysis for the purpose of this study.

### Statistical analysis

Demographic information is described as median (IQR) or absolute (relative) frequency. All plasma laboratory values after arginine administration are described by median (IQR) for baseline, 60 min, 90 min, and 120 min.

Correlations between urea and copeptin levels were assessed by calculating Spearman’s rank correlation coefficient. A correlation coefficient (Spearman’s rho) of 0.00–0.40 was considered a weak correlation, 0.41–0.60 as moderate, and 0.61–1.00 a strong correlation [13]. Correlations were calculated for each timepoint (i.e., baseline, 60 min, 90 min, and 120 min) and for the changes in urea and copeptin levels (∆urea, ∆copeptin) at timepoints 60 min, 90 min, and 120 min.

Additionally, all correlations were recalculated in a sensitivity analysis, excluding participants with a baseline plasma osmolality < 285, as this may introduce confounding effects on copeptin.

Urea, osmolality, sodium, glucose, and copeptin dynamics upon arginine infusion are visually presented with boxplots. Data between the groups was compared visually and by assessing summary statistics (median, IQR). All analyses are exploratory, and no hypothesis testing was performed. Following recent recommendations, the term “statistically significant” is not used [14]. Missing data was not imputed. All analyses were performed in R version 4.2.3.

## Results

### Baseline characteristics

In total 30 HA, 69 patients with AVP-D, and 89 with PP were included. The median age was 27 years (24, 34) for HA, 42 years (32, 54) for patients with AVP-D and 37 years (28, 50) for patients with PP. In all three groups, the majority of participants was female with similar percentages in HA (57%) and patients with AVP-D (55%), while being distinctly higher in patients with PP (76%). Median body mass index (BMI) was in the normal range for HA (23.3 kg/m^2^ (20.5, 26.1)) and patients with PP (23.8 kg/m^2^ (21.0, 28.6)) and in the overweight range for patients with AVP-D (27.7 kg/m^2^ (24.5, 33.0)). Baseline characteristics for each group and information on the cause of AVP-D are presented in Table 1.

**Table 1:** Baseline characteristics

	HA	AVP-D	PP
n	30	69	89
Age (years)	27 (24, 34)	42 (32, 54)	37 (28, 50)
Sex (% female)	17 (57)	38 (55)	68 (76)
Weight (kg)	67 (60, 80)	85 (69, 99)	68 (58, 84)
Body mass index (kg/m^2^)	23.3 (20.5, 26.1)	27.7 (24.5, 33.0)	23.8 (21.0, 28.6)
Systolic blood pressure (mm Hg)	118 (108, 123)	125 (114, 137)	117 (110, 130)
Diastolic blood pressure (mm Hg)	71 (67, 77)	78 (70, 85)	70 (65, 80)
eGFR (mL/min/1.73 m^2^)	102 (89, 115)	90 (81, 110)	97 (87, 110)
Copeptin (pmol/L)	5.8 (4.5, 11.6)	2.3 (1.8, 3.0)	2.7 (2.2, 4.2)
Urea (mmol/L)	4.4 (3.8, 5.3)	3.9 (2.6, 4.7)	3.7 (3.0, 4.7)
Sodium (mmol/L)	141 (139, 142)	143 (142, 145)	140 (139, 141)
Glucose (mmol/L)	4.9 (4.8, 5.5)	5.1 (4.8, 5.4)	5.0 (4.6, 5.2)
Plasma Osmolality (mOsm/kg)	292 (289, 295)	292 (289, 295)	285 (281, 289)
Urine Osmolality (mOsm/kg)	789 (630, 901)	181 (108, 298)	222 (156, 431)
Cause of AVP-D			
Postsurgical condition	–	21 (30)	–
Hypothalamic-pituitary lesions	–	18 (26)	–
Trauma	–	5 (7)	–
Empty sella or hypoplasia	–	5 (7)	–
Vascular	–	1 (1)	–
Hypophysitis	–	8 (12)	–
Idiopathic	–	8 (12)	–
Familial	–	3 (4)	–

Data is presented as median (IQR) or n (%)

*HA* healthy adults, *AVP-D* patients with arginine vasopressin deficiency, *PP* patients with primary polydipsia, *eGFR* estimated glomerular filtration rate

### Plasma urea, osmolality, and copeptin dynamics upon arginine stimulation

**Fig. 1:**
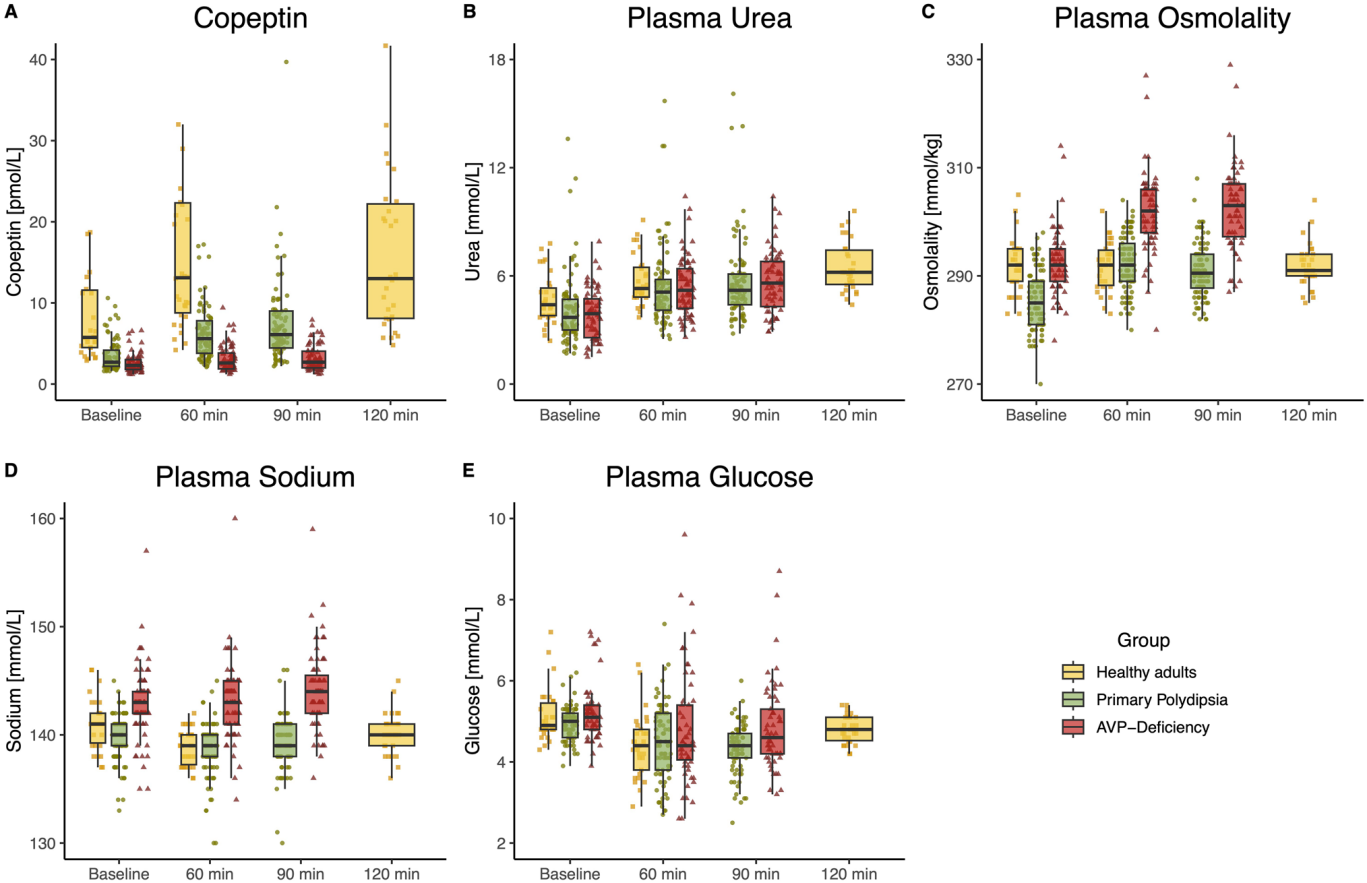
Effects of arginine infusion in healthy adults, patients with primary polydipsia and patients with arginine vasopressin deficiency (AVP-Deficiency). The overlaying scatterplot shows the individual data points for each patient. Boxes span the interquartile range (IQR); the thick horizontal line is the median. Whiskers are the most extreme values lying within the box edge and 1.5 times the IQR. Presented are **A** Copeptin levels (15 Copeptin values > 42 pmol/L are not shown), **B** Plasma Urea levels, **C** Plasma Osmolality (1 osmolality value < 270 mOsm/kg is not shown), **D** Plasma Sodium levels, **E** Plasma Glucose levels at baseline, 60, 90 and 120 min upon arginine infusion

The time course of plasma urea, osmolality, and copeptin after arginine infusion is shown in Fig. 1 for HA and for patients with AVP-D and PP. Changes in plasma urea, osmolality, and copeptin levels from baseline (i.e., immediately before the start of arginine infusion) to the end of the test (i.e., 90 or 120 min) are demonstrated in Table 2.

**Table 2:** Timecourse of urea, copeptin, osmolality, sodium, and glucose upon arginine infusion

	HA	AVP-D	PP
	*Baseline*		
Urea (mmol/L)	4.4 (3.8, 5.3)	3.9 (2.6, 4.7)	3.7 (3.0, 4.7)
Copeptin (pmol/L)	5.8 (4.5, 11.6)	2.3 (1.8, 3.0)	2.7 (2.2, 4.2)
Plasma Osmolality (mOsm/kg)	292 (289, 295)	292 (289, 295)	285 (281, 289)
Sodium (mmol/L)	141 (139, 142)	143 (142, 144)	140 (139, 141)
Glucose (mmol/L)	4.9 (4.8, 5.5)	5.1 (4.8, 5.4)	5.0 (4.6, 5.2)
	*60 min*		
Urea (mmol/L)	5.3 (4.8, 6.5)	5.2 (4.2, 6.4)	5.1 (4.1, 5.8)
∆ Urea (mmol/L)	+ 1.1 (+ 0.8, + 1.5)	+ 1.4 (+ 1.1, + 1.7)	+ 1.3 (+ 0.9, + 1.5)
Copeptin (pmol/L)	13.1 (8.8, 22.3)	2.6 (1.9, 3.8)	5.6 (3.8, 7.8)
∆ Copeptin (pmol/L)	+ 5.3 (+ 3.2, + 8.8)	+ 0.3 (+ 0.1, + 0.6)	+ 2.4 (+ 1.2, + 3.8)
Plasma Osmolality (mOsm/kg)	292 (288, 295)	302 (298, 306)	292 (289, 296)
∆ Plasma Osmolality (mOsm/kg)	+ 1 (− 4, + 3)	+ 10 (+ 7, + 12)	+ 7 (+ 5, + 9)
Sodium (mmol/L)	139 (137, 140)	143 (141, 145)	139 (138, 140)
∆ Sodium (mmol/L)	− 3 (− 4, ± 0)	± 0 (− 1, + 1)	− 1 (− 2, ± 0)
Glucose (mmol/L)	4.4 (3.8, 4.8)	4.4 (4.1, 5.4)	4.5 (3.8, 5.2)
∆ Glucose (mmol/L)	− 0.7 (− 1.1, − 0.2)	− 0.5 (− 1.1, − 0.1)	− 0.5 (− 1.1, + 0.1)
	*90 min*		
Urea (mmol/L)	–	5.6 (4.3, 6.8)	5.2 (4.4, 6.1)
∆ Urea (mmol/L)	–	+ 1.7 (+ 1.3, + 2.1)	+ 1.5 (+ 1.2, + 1.9)
Copeptin (pmol/L)	–	2.7 (2.0, 4.1)	6.1 (4.5, 9.0)
∆ Copeptin (pmol/L)	–	+ 0.4 (± 0.0, + 0.7)	+ 2.7 (+ 1.6, + 4.9)
Plasma osmolality (mOsm/kg)	–	303 (297, 307)	291 (288, 294)
∆ Plasma osmolality (mOsm/kg)	–	+ 10 (+ 6, + 13)	+ 6 (+ 4, + 8)
Sodium (mmol/L)	–	144 (142, 146)	139 (138, 141)
∆ Sodium (mmol/L)	–	+ 1 (± 0, + 3)	− 1 (− 2, + 1)
Glucose (mmol/L)	–	4.6 (4.2, 5.3)	4.4 (4.1, 4.7)
∆ Glucose (mmol/L)	–	− 0.6 (− 0.9, ± 0.0)	− 0.5 (− 0.9, − 0.2)
	*120 min*		
Urea (mmol/L)	6.2 (5.5, 7.4)	–	–
∆ Urea (mmol/L)	+ 1.8 (+ 1.5, + 2.1)	–	–
Copeptin (pmol/L)	13.0 (8.1, 22.2)	–	–
∆ Copeptin (pmol/L)	+ 5.0 (+ 2.7, + 8.9)	–	–
Plasma osmolality (mOsm/kg)	291 (290, 294)	–	–
∆ Plasma osmolality (mOsm/kg)	+ 1 (− 1, + 2)	–	–
Sodium (mmol/L)	140 (139, 141)	–	–
∆ Sodium (mmol/L)	− 1 (− 2, ± 0)	–	–
Glucose (mmol/L)	4.8 (4.5, 5.1)	–	–
∆ Glucose (mmol/L)	− 0.1 (− 0.4, + 0.1)	–	–

Data is presented as median (IQR)

*HA* healthy adults, *AVP-D* patients with arginine vasopressin deficiency, *PP* patients with primary polydipsia

#### Urea

Baseline plasma urea levels were similar for all three groups: 4.4 mmol/L (3.8, 5.3) for HA, 3.9 mmol/L (2.6, 4.7) for patients with AVP-D and 3.7 mmol/L (3.0, 4.7) for patients with PP. After 60 min, urea levels increased similarly in all groups, with a median increase (∆ urea) of + 1.1 mmol/L (+ 0.8, + 1.5) for HA, + 1.4 mmol/L (+ 1.1, + 1.7) for patients with AVP-D and + 1.3 mmol/L (+ 0.9, + 1.5) for patients with PP. The test period ended after 90 min for patients with AVP-D and patients with PP, and after 120 min for HA. Median urea levels raised to a maximum of 5.6 mmol/L (4.3, 6.8) in patients with AVP-D and 5.2 mmol/L (4.4, 6.1) in PP after 90 min. Median urea levels in HA reached a maximum of 6.2 mmol/L (5.5, 7.4) after 120 min.

#### Osmolality

Baseline plasma osmolality was similar in HA with 292 mOsm/kg (289, 295) and patients with AVP-D with 292 mOsm/kg (289, 295), while it was lower in patients with PP with 285 mOsm/kg (281, 289). In HA, osmolality remained stable throughout the test, with a median change compared to baseline of + 1 mOsm/kg (− 4, + 3) at 60 min and + 1 mOsm/kg (− 1, + 2) at 120 min. In contrast, both patients with AVP-D and PP showed an increase in osmolality: for AVP-D, the median increase compared to baseline was + 10 mOsm/kg (+ 7, + 12) at 60 min and + 10 mOsm/kg (+ 6, + 13) at 90 min, resulting in a median osmolality of 303 mOsm/kg (297, 307) at the end of the test. For PP, median osmolality increased + 7 mOsm/kg (+ 5, + 9) at 60 min and + 6 mOsm/kg (+ 4, + 8) at 90 min, with a corresponding median osmolality of 291 mOsm/kg (288, 294) at the end of the test.

#### Copeptin

Median copeptin levels at baseline were highest in HA with 5.8 pmol/L (4.5, 11.6). Patients with AVP-D and PP showed lower levels at baseline with a median of 2.3 pmol/L (1.8, 3.0) and 2.7 pmol/L (2.2, 4.2) respectively. Copeptin levels increased during the test period in all three groups, with the highest median levels of 13.1 pmol/L (8.8, 22.3) seen in HA after 60 min. Those levels remained stable until the end of the test. In patients with PP the highest median copeptin levels of 6.1 pmol/L (4.5, 9.0) were reached after 90 min. Median copeptin levels of patients with AVP-D showed only a minimal increase over time, with the maximal levels of 2.7 pmol/L (2.0, 4.1) reached after 90 min.

The median increase in maximal copeptin levels (∆ copeptin) was greatest in HA with + 5.3 pmol/L (+ 3.2, + 8.8) after 60 min, followed by patients with PP with + 2.7 pmol/L (+ 1.6, + 4.9) after 90 min, and only marginal in patients with AVP-D with + 0.4 pmol/L (± 0.0, + 0.7) after 90 min.

### Correlation between plasma urea and copeptin levels

Correlations of plasma urea and copeptin levels, and changes in plasma urea and copeptin levels for each timepoint are presented in Table 3.

**Table 3:** Correlations of urea and copeptin plasma levels

	Full data set analysis		Sensitivity analysis (baseline osmolality > 285 mOsm/kg)	
n	HA	30	HA	28 (93%)
	AVP-D	69	AVP-D	64 (93%)
	PP	89	PP	47 (53%)
	Correlation of Copeptin and urea plasma levels (Spearman’s rho)	Correlation of ∆ Copeptin and ∆ Urea (Spearman’s rho)	Correlation of Copeptin and urea plasma levels (Spearman’s rho)	Correlation of ∆ Copeptin and ∆ Urea (Spearman’s rho)
	Baseline			
HA	0.41	–	0.45	–
AVP-D	0.06	–	0.17	–
PP	0.28	–	0.31	–
	60 min			
HA	0.24	0.08	0.22	0.04
AVP-D	0.14	0.21	0.10	0.17
PP	0.22	0.22	0.21	0.23
	90 min			
HA	–	–	–	–
AVP-D	0.01	0.16	− 0.05	0.18
PP	0.22	0.24	0.21	0.17
	120 min			
HA	0.40	0.35	0.42	0.32
AVP-D	–	–	–	–
PP	–	–	–	–

*HA* healthy adults, *AVP-D* patients with arginine vasopressin deficiency, *PP* patients with primary polydipsia

The correlation of plasma urea and plasma copeptin was strongest in HA, with a Spearman’s rho of 0.41 at baseline, 0.24 at 60 min and 0.40 at 120 min, corresponding to a weak to moderate correlation. Patients with AVP-D showed overall weak correlations of urea and copeptin levels, with the greatest correlation coefficient of 0.28 observed at baseline, remaining stable throughout the test. Patients with PP showed the weakest correlation of plasma urea and copeptin, with a correlation ranging from 0.01 at 90 min to 0.14 at 60 min.

Similarly, the changes of urea and copeptin levels (∆urea and ∆copeptin) showed only a weak correlation, with the greatest correlation seen in HA at 120 min with a Spearman’s rho of 0.35. The correlation of the changes observed at the timepoint of the greatest increase in copeptin was 0.08 in HA at 60 min, 0.16 in patients with PP at 90 min, and 0.24 in patients with AVP-D at 90 min.

In a sensitivity analysis, subjects with baseline plasma osmolality < 285 mmol/L were excluded. The number of observations in the sensitivity analysis set compared to the full analysis set were 28 of 30 for HA, 47 of 89 for patients with PP and 64 of 69 for AVP-D. When comparing the correlations between the two datasets, no relevant changes were observed (Table 3).

## Discussion

Our study has two main findings. First, we demonstrate a slight increase in plasma urea levels in response to arginine in all three groups, with a substantial increase in plasma osmolality in patients with AVP-D and with PP but stable levels in HA. The conversion of arginine in the liver into ornithine and urea allows the body to eliminate toxic ammonia [10]. The surplus of arginine provided by the study infusion rapidly increases the rate of conversion, as shown previously [15], and is therefore likely responsible for the elevated levels of plasma urea seen in all three groups. Second, we observed only weak correlations, both for the absolute levels of plasma urea and copeptin and for the changes during the test. Based on these findings, the stimulatory effect of arginine on AVP is unlikely to be explained primarily by increasing urea levels.

The only weak correlation between plasma urea and copeptin levels stands in contrast to our initial hypothesis. Indeed, a direct central stimulation of arginine-stimulated urea driven by an increase in plasma osmolality seems unlikely. Potent stimuli to AVP release are increasing plasma osmolality and unspecific stimuli, such as nausea, pain, or hypoglycemia [16, 17]. Hypothalamic magnocellular neurons are involved in osmoregulation and particularly sensitive to changes in concentrations of osmotically active solutes [17–19]. In 1983, Zerbe and colleagues demonstrated that in HA, infusion of hypertonic saline increases plasma osmolality, leading to a strong elevation of AVP plasma levels with a strong positive correlation between AVP and osmolality. While intravenous hypertonic urea led to a strong increase in plasma urea and plasma osmolality, it induces a delayed and less pronounced rise in AVP [12]. The changes in plasma urea and osmolality upon urea infusion were much greater than what we observed in our study, thus supporting our conclusion of urea playing only a minor role in arginine-mediated stimulation of copeptin. Further, these observations suggest that the osmoreceptors are more sensitive to sodium than urea, probably due to their different diffusion potential: while sodium hardly crosses the cell membrane due to its electric charge and the lack of transporters, urea, though hydrophilic, may enter the cell through urea transporters nearly as fast as water and is therefore considered an ‘ineffective osmole’ [20]. Consequently, the difference in osmotic pressure between the blood and cell fluid is less pronounced in urea-induced hyperosmolality, leading to diminished cell shrinkage compared to sodium-induced hyperosmolality, resulting in a lower AVP secretion. Our findings suggest a similar plasma urea dynamic in response to arginine in all groups with however different copeptin and osmolality response. In HA, with increasing urea levels increasing copeptin (i.e. vasopressin) can be observed and plasma osmolality maintains within the physiological range. In patients with AVP-D this function may be impaired and a more pronounced rise of plasma osmolality into a state of hyperosmolality can be observed. In PP, most likely the lower baseline plasma osmolality leads to less pronounced vasopressin release and therefore also to the observed increase of plasma osmolality. However, one should note that the pronounced increase in osmolality in patients cannot be explained solely by the mild increase in urea levels.

In our study, no data on urine urea and changes in its fractional excretion were available. However, possible peripheral effects of urea in the kidney may be considered: increased urea excretion creates an osmotic effect in the renal tubules, and as a result, more water is excreted, leading to increased urine output. The release of AVP stimulates the insertion of aquaporin 2 channels, counteracting this free water loss [19]. In addition, AVP also increases the insertion of urea transporters into the luminal membrane of the collecting duct cells [21]. These urea transporters facilitate the reabsorption of urea from the urine back into the cells and contribute to the high concentration of solutes in the medullary interstitium, creating an osmotic gradient that contributes to the aquaporin 2-induced reabsorption of free water [21]. However, it is important to note that this effect is not likely to provide a full understanding of AVP release in response to arginine, and alternative explanations for the stimulative effect must be considered.

While urea levels increased in all three observed groups, plasma osmolality only increased substantially in AVP-D and PP. HA and AVP-D already started the test with a relatively high plasma osmolality, but while HA showed only a marginal increase, patients with AVP-D exceeded the threshold of normal osmolality at 300 mOsm/kg within 60 min and remained hyperosmolar until the end of the test. The increase in osmolality in AVP-D was expected and arises from the patients not being allowed to drink throughout the test in combination with the lack of AVP effect, resulting in the loss of free water. Plasma osmolality in PP at baseline was substantially lower than in HA and patients with AVP-D but later rose to similar levels as HA during the test. This trend can be considered as part of the physiologic adaptation to fluid restriction. The increase in urea levels may explain the marginal rise of plasma osmolality in HA but may only be partly responsible for the pronounced elevation of plasma osmolality in AVP-D and PP.

Overall, evidence from this anaylsis suggest that the stimulatory effect of arginine on AVP secretion is most likely a combination of various mechanisms: First, arginine is a precursor for the synthesis of nitric oxide (NO) catalyzed by NO synthases (NOS) present in various cell types, including hypothalamic AVP-producing neurons [22–25]. This mechanism involves arginine being converted into L-citrulline by NOS, with NO being produced as a byproduct. NO directly stimulates AVP-producing neurons in the hypothalamus [23] and administering NO donors such as arginine could therefore lead to AVP release. Second, hypoglycaemia is one of the most potent non-osmotic stimuli for the pituitary gland and induces an AVP release [26]. Therefore, glucose dynamics upon arginine infusion could offer another explanation as a distinct glucose pattern can be observed, characterized by an initial increase at 30 min followed by a decrease to low-normal values, but without leading to absolute hypoglycaemia [27]. This is attributed to arginine’s ability to depolarize pancreatic beta cells resulting in increased insulin secretion [28] and the subsequent glucose drop might trigger non-osmotic AVP release [27]. However, this mechanism is controversial [29] and one should note the moderate median glucose drop of 1.9 mmol/l observed upon arginine. Third, physiological studies in humans and animals have demonstrated that the magnocellular-neurohypophyseal system can be activated with nausea-inducing agents [30–32]. Although nausea was a frequent symptom during arginine stimulation, it was generally mild and of transient nature. Interestingly, basal nausea levels were not associated with elevated copeptin [7] and therefore only more severe nausea, or vomiting should be considered as potential strong stimuli [31, 33, 34]. Finally, several pituitary stimulation tests leading to growth hormone (GH) stimulation (e.g., arginine, insulin, or glucagon) also lead to copeptin stimulation [35, 36]. One could assume that GH might directly stimulate vasopressin. However, our previous study on macimorelin contradicts this hypothesis [37]. The GH course upon arginine, insulin, or glucagon stimulation is very similar to the copeptin course. In contrast, while a strong GH increase was observed upon ingestion of macimorelin, no change in copeptin was shown. We rather speculate that for e.g., insulin or glucagon, the simultaneous copeptin and GH increase arises from a common stimulus, rather than a direct stimulatory effect of GH on vasopressin.

Our study has limitations. The main limitation is that this is a post-hoc analysis of two prospective studies, limiting further sub-group analysis. The test protocol differed between the two studies in the test duration (90 min for patients with PP and AVP-D, 120 min for HA), complicating the comparison between the results at the end of the test. In general, it is more likely to miss correlations between analytes with only a few observations specifically in a dynamic test setting, especially due to the lack of data between baseline and 60 min. Further, possible confounding factors such as the differences in sex and age between the groups, low plasma osmolality at the beginning of the test or non-osmotic effects of arginine, such as nausea, could have skewed the actual effect.

In conclusion, this analysis shows a slight increase in plasma urea and a weak correlation between plasma copeptin and urea in response to arginine infusion, suggesting that the effect on copeptin cannot be primarily explained by this mechanism. The stimulatory effect of arginine on AVP secretion is most likely a combination of various mechanisms, with increased urea synthesis playing a minor role in the overall process.

## Data Availability

The datasets and code used for this analysis will be made available upon request.
